# A Cadaveric Case Report of a Rare Atrial Septal Lipoma

**DOI:** 10.7759/cureus.71794

**Published:** 2024-10-18

**Authors:** Hannah J Grimmett, Ketsia Kimbimbi, Ava Greenberg, Niharika Dar, Hadiseh Faridi Tavana, Kamal A Abouzaid, Ahmad Imam

**Affiliations:** 1 Department of Anatomical Sciences, William Carey University College of Osteopathic Medicine, Hattiesburg, USA

**Keywords:** atrial septal lipoma, cadaveric case report, cardiac mass, dilated right atrium, lipoma, primary cardiac tumor, subendocardial lipoma

## Abstract

Primary cardiac tumors (PCTs) are rare and include myxomas, papillary fibroelastomas, rhabdomyomas, and lipomas. Lipomatous hypertrophy of the atrial septum (LHAS) is a benign condition associated with aging and obesity that is marked by fat accumulation in the interatrial septum and is caused by hyperplasia of adipose cells. In contrast, lipomas are characterized as soft masses of fat that are encapsulated by thin, fibrous tissue. True cardiac lipomas are rare and can present a diagnostic dilemma. Cardiac lipomas are generally asymptomatic but may present with angina, dyspnea, or syncope. Less frequently, they can cause arrhythmias, valve dysfunction, or emboli. Diagnosis generally requires cardiac MRI or alternate imaging modality.

In this report, we present a case of a true atrial septal lipoma with a sessile base protruding into the right atrium discovered during the pedagogic dissection assignment of the interior of the heart. The tumor measured 0.5 × 1.0 cm, and on the cut surface, it appeared yellow and encapsulated. Histopathological examination of the lipomatous mass revealed extensive nodular thickening of the interatrial septum from the accumulation of mature adipose tissue reaching the resection surface. It is possible that the lipoma may have had a space-occupying effect, which would have increased the volume within the right atrium and had an adverse effect on tricuspid valve function. This is consistent with the dilated and thin-walled appearance of the right atrium. This report contributes to the limited literature on this type of benign, primary cardiac tumor and provides a clear illustration and clinical relevance to showcase the pathology and its possible clinical consequences.

## Introduction

Primary cardiac tumors (PCTs) are a rare occurrence with an incidence range of 0.0017%-0.03% on postmortem examination [1]. All PCTs are of mesodermal origin with the exception of tumors of misplaced endodermal tissue and neural tumors that originate from ectoderm [2]. Lipomatous hypertrophy of the atrial septum (LHAS) is a benign condition marked by fat accumulation in the interatrial septum [3]. It is characterized by the accumulation of fat in the interatrial septum of more than 2 cm in thickness [4]. This occurs due to the hyperplasia of adipose cells and is associated with aging and obesity [5]. Microscopically, it presents a combination of adipose tissue and myocytes [3]. In contrast, lipomas can be defined as masses of fat that are enclosed by a capsule of fibrous tissue [6]. They account for 8.4% of benign PCT; they are the second most common PCT [7]. Additionally, lipomas have an equal incidence rate in both genders and can occur at any age [8]. However, approximately 60% of lipomas occur between the ages of 40 and 70 [6]. Burke and Virmani studied 386 PCTs; 12 of the cases were lipomatous hypertrophy of the atrial septum (LHAS) (3%), and two of the cases were true lipomas (0.005%) [2]. The aforementioned study underscores the rarity of a true cardiac lipoma.

All three layers of cardiac tissue can give rise to lipomas; 50% of cardiac lipomas occur in the subendocardium, while the remaining 50% is equally distributed between the subpericardium and myocardium [8]. Intramyocardial fat can be subdivided into subendocardial and non-subendocardial fat. Subendocardial fat is mostly found in the left ventricle myocardium within a post-myocardial infarction (MI) scar, and it is typically seen in elderly or middle-aged males [9]. Shu et al. examined 255 cases with cardiac lipomas, 38% were found incidentally with 29.8% during a routine checkup, 5.9% at autopsy following an unrelated death, and 2.4% during an unrelated surgery. Out of the 255 cases, 42.4% were located in the right atrium [6].

Lipomas are generally benign; however, based on their location, they may produce symptoms. The majority appear in the subendocardium, but they can also appear in the subepicardium and within the myocardium [10]. Subendocardial lipomas tend to be small and sessile, while subepicardial lipomas can grow larger, potentially causing anginal pain due to external compression of the coronary arteries [11].

In this case report, we observed and described a very rare true atrial septal lipoma with a sessile base protruding into the right atrium. Our findings contribute to the limited literature on this type of benign, primary cardiac tumor and provide a clear description and photographic depiction to showcase the pathology and its possible effects on the right atrium.

## Case presentation

A rare case of cardiac lipoma was observed during the pedagogic dissection assignment of the interior of the heart for first-year medical students at William Carey University College of Osteopathic Medicine. The cadaveric donor was a 74-year-old female Caucasian and was obtained from the University of Southern Alabama Anatomical Gift Program. The cause of death, as documented in the death certificate, was parkinsonism.

**Figure 1:**
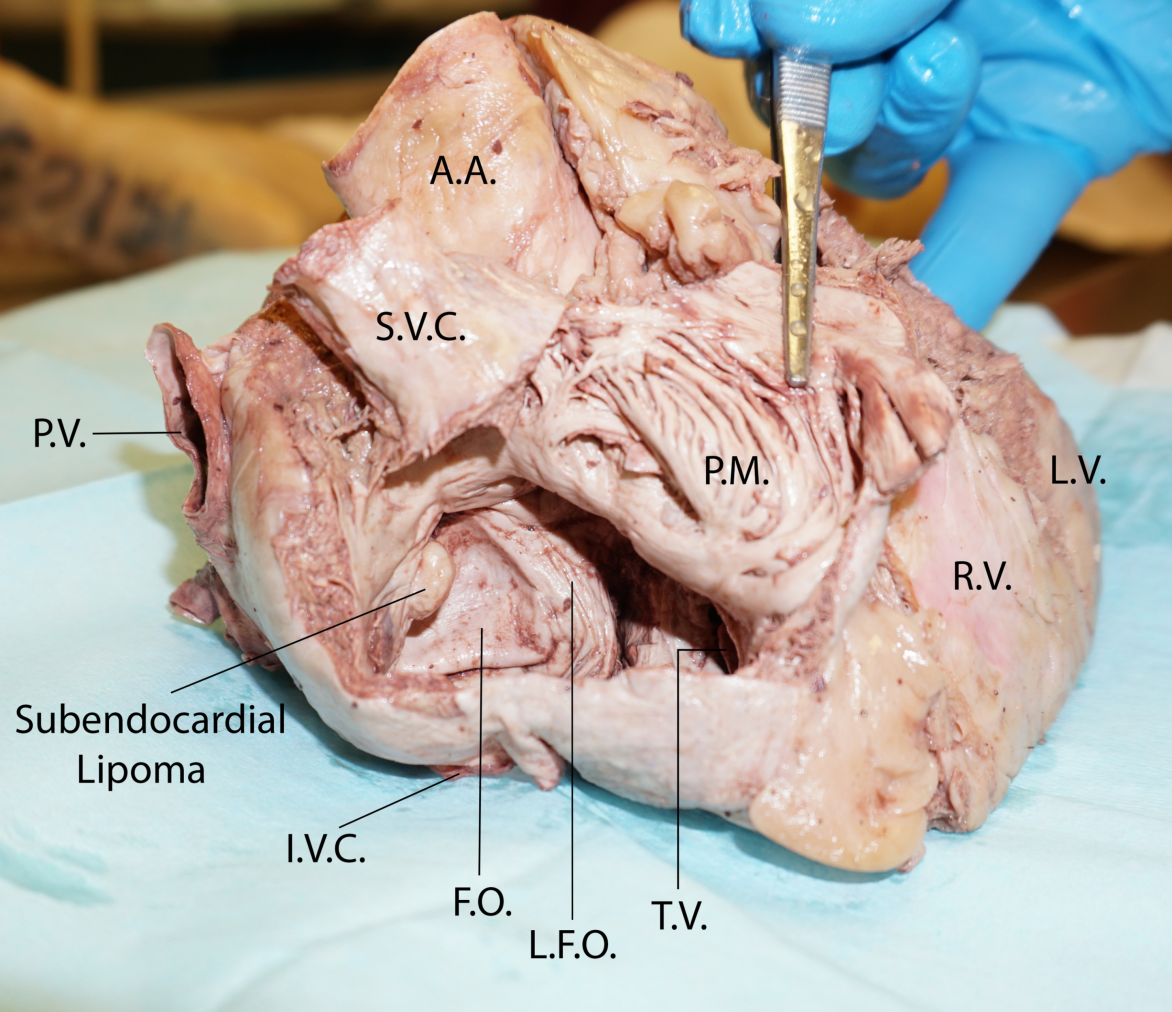
Anterior view of the heart: the anterior wall of the right atrium was open and retracted to the left to show the interatrial septum and the subendocardial lipoma RV: right ventricle, LV: left ventricle, PM: pectinate muscles, TV: tricuspid valve, IVC: inferior vena cava, SVC: superior vena cava, FO: fossa ovalis, LFO: limbus fossa ovalis, AA: ascending aorta, PA: pulmonary artery, PV: pulmonary vein

To open the right atrial chamber, a transverse incision was made through the free edge of the right auricle close to its superior border. The incision was advanced toward the right up to the level of the opening of the superior vena cava. From the right extent of the transverse incision, a second vertical incision was made at the right edge of the right atrium and extended inferiorly to the level of the opening of the inferior vena cava. A second transverse incision was made from the lower extent of the vertical incision and was extended for a short distance toward the coronary sulcus. The flap of the anterior wall of the right atrium was retracted to the left, and the interior of the right atrium was cleaned from blood clots to examine the internal features of the chamber. The right atrium appeared dilated and thin-walled. Examination of the interatrial septal wall revealed a soft tissue tumor arising from the right margin of the limbus fossa ovalis and protruding into the right atrium (Figure 1).

**Figure 2:**
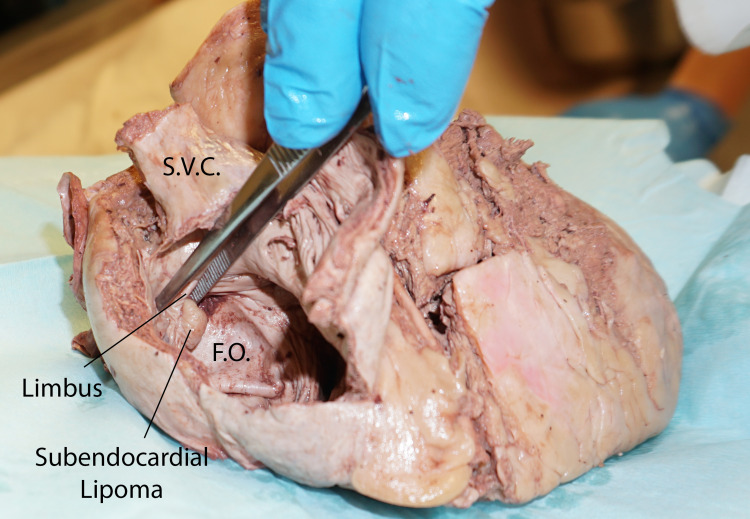
Close-up view of the interior of the right atrium showing the interatrial septum and the subendocardial lipoma arising from the right margin of the limbus fossa ovalis SVC: superior vena cava, FO: fossa ovalis

**Figure 3:**
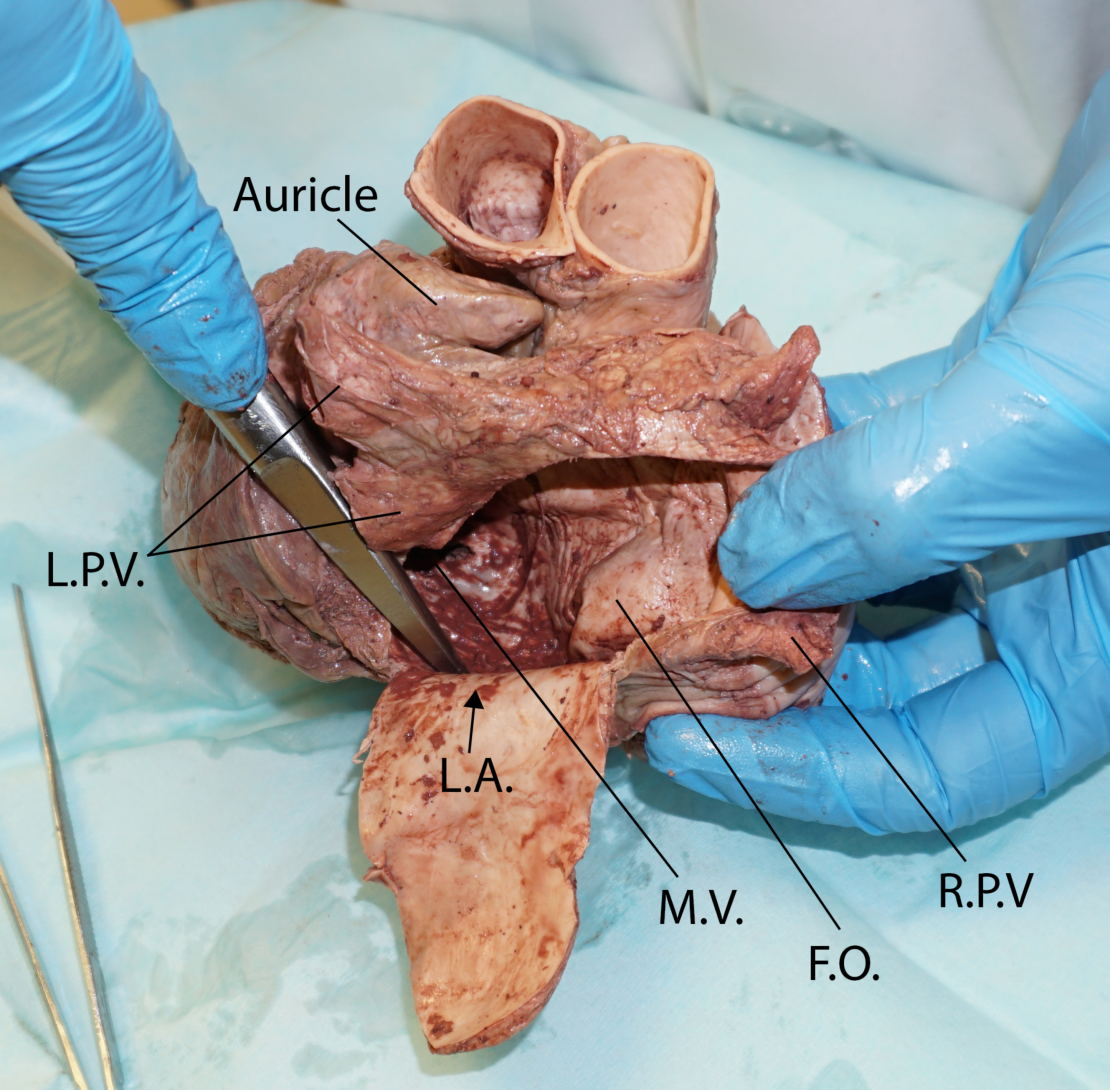
Posterior view of the base of the heart showing the interior of the left atrium: the fossa ovalis is protruding into the left atrium LA: left atrium, FO: fossa ovalis, MV: mitral valve, LPV: left pulmonary veins, RPV: right pulmonary veins

The tumor was subendocardial and yellow in color and had a wide base. The fossa ovalis was thin and ballooned out into the left atrium (Figure 2 and Figure 3).

**Figure 4:**
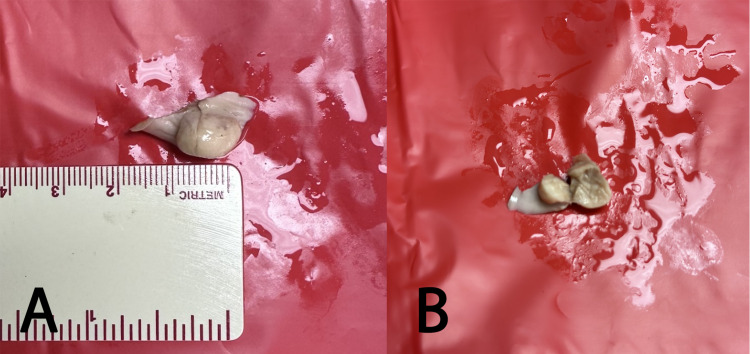
The lipoma was excised and measured (A); it was incised to show its cut surface (B)

The left atrium was also opened using an inverted U-shaped incision between the pulmonary veins. The tumor was not seen when examining the septal wall from the left atrial side. The tumor was excised with the septal wall and incised to examine the internal features. The tumor measured 0.5 × 1.0 cm, and on the cut surface, it appeared yellow and encapsulated (Figure 4).

**Figure 5:**
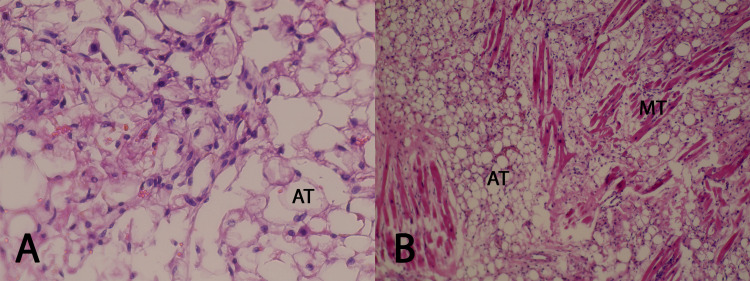
Histopathological examination of the resected mass revealed lipomatous hypertrophy of the interatrial septum with no signs of malignancy (A: H&E staining, magnification: × 400; B: H&E staining, magnification: ×200) AT: adipose tissue, MT: muscular tissue, H&E: hematoxylin and eosin

Histopathological examination of the lipomatous mass revealed extensive nodular thickening of the interatrial septum from the accumulation of mature adipose tissue reaching the resection surface (Figure 5). At higher magnification, fatty tissue was dissected by residual muscle fibers recognized by cellular characteristics. Moreover, occasional nuclear enlargement was seen, but no atypia. Additionally, diffuse deposition of lipofuscin was seen throughout the specimen.

## Discussion

Cardiac lipomas may be symptomatic or asymptomatic and found incidentally during a diagnostic procedure for an unrelated ailment [12]. The location and size of the tumor determine the severity of the symptoms. For instance, a myocardial lipoma would have different symptoms compared to a subendocardial lipoma [10]. Although patients with cardiac lipomas can remain asymptomatic for some time, larger subepicardial lipomas may compress the coronary arteries and cause anginal pain, dyspnea, arrhythmias, and embolisms [11]. Due to the fact that lipomas are typically encapsulated, embolization is a rare complication [10]. These benign tumors may also cause valve dysfunction, flow obstruction, and eventually cardiac failure [11]. Excessive growth of a lipoma and penetration into the myocardium could suggest a more serious clinical manifestation and an unfavorable prognosis [6,13]. In our case, the right atrium appeared dilated and thin-walled. The atrial dilation is suggestive of chronic hemodynamic overload, which is a precursor to heart failure [14]. Subendocardial benign tumors may cause obstruction of blood flow [15]. One can argue that the lipoma seen in the present case had a space-occupying effect, which would have increased the volume within the right atrium and might have had an adverse effect on tricuspid valve function. Moreover, it might have led to obstruction of the blood flow into the right ventricle. Atrial enlargement is more commonly a result of persistent atrial fibrillation or volume overload triggered by predisposing conditions such as right ventricular disease, tricuspid valve disease, congenital heart anomalies, or pulmonary hypertension [16].

PCTs have been shown to be associated with ventricular tachycardia (VT); VT occurs due to the localized electrical re-entry at the tumor margin [17]. In pediatric patients with primary cardiac tumors, four clinically significant arrhythmias were found: sudden cardiac arrest with documented or suspected ventricular fibrillation, non-sustained and sustained VT, pre-excitation, and sustained supraventricular tachycardia of any mechanism with VT being the most common type. Of all PCTs, lipomas are less likely to cause arrhythmias, but they can still elicit other symptoms [18]. They may cause fatigue and syncope regardless of whether the mass is considered a true lipoma or LHAS [19]. In one report, shortness of breath was the only relevant symptom of a patient with an endocardial lipoma [10]. In our case report, we observed that the septal wall was ballooned into the left atrium, which may have decreased the volume of the left atrium, resulting in pulmonary congestion; this would explain symptoms such as shortness of breath, fatigue, and syncope. Despite the potential for severe complications, to our knowledge, there is no established screening protocol for cardiac tumors. The preferred imaging technique for the diagnosis of a cardiac lipoma is MRI; however, echocardiography, CT scan, angiography, and radiography are also appropriate [2].

## Conclusions

A high percentage of PCTs have been found at autopsy, which indicates underdiagnosis of these conditions. This is most likely due to the non-specific nature of the symptoms and signs relevant to these masses. This report presents a rare case of atrial septal subendocardial lipoma, which adds to the limited literature on true cardiac lipomas. Furthermore, we described our observations in detail and discussed the potential pathophysiological effects and clinical implications. Although rare, cardiac tumors should always be in the differential diagnosis in patients with unexplained cardiac conditions.
